# Vascular Cell Co-Culture on Silk Fibroin Matrix

**DOI:** 10.3390/polym10010039

**Published:** 2018-01-01

**Authors:** Fangfang Tu, Yunfei Liu, Helei Li, Pange Shi, Yunxia Hao, Yue Wu, Honggen Yi, Yin Yin, Jiannan Wang

**Affiliations:** 1 College of Textile and Clothing Engineering, Soochow University, Suzhou 215123, Jiangsu, China; 20154215025@stu.suda.edu.cn (F.T.); 20154215023@stu.suda.edu.cn (Y.L.); 20164215017@stu.suda.edu.cn (H.L.); 20165215003@stu.suda.edu.cn (P.S.); yunxiahao132820@163.com (Y.H.); 20154215026@stu.suda.edu.cn (Y.W.); 2 National Engineering Laboratory for Modern Silk, Soochow University, Suzhou 215123, Jiangsu, China; yihg@suda.edu.cn; 3 Laboratory Animal Research Center, Soochow University, Suzhou 215123, Jiangsu, China; szwwh082@gmail.com

**Keywords:** **Keywords**: vascular cells, co-culture, silk fibroin, proliferative activity, cells interaction

## Abstract

Silk fibroin (SF), a natural polymer material possessing excellent biocompatibility and biodegradability, and has been widely used in biomedical applications. In order to explore the behavior of vascular cells by co-culturing on regenerated SF matrix for use as artificial blood vessels, human aorta vascular smooth muscle cells (HAVSMCs) were co-cultured with human arterial fibroblasts (HAFs) or human umbilical vein endothelial cells (HUVECs) on SF films and SF tubular scaffolds (SFTSs). Analysis of cell morphology and deoxyribonucleic acid (DNA) content showed that HUVECs, HAVSMCs and HAFs adhered and spread well, and exhibited high proliferative activity whether cultured alone or in co-culture. Immunofluorescence and scanning electron microscopy (SEM) analysis showed that HUVECs and HAFs co-existed well with HAVSMCs on SF films or SFTSs. Cytokine expression determined by reverse transcription-polymerase chain reaction (RT-PCR) indicated that the expression levels of α-smooth muscle actin (α-SMA) and smooth muscle myosin heavy chain (SM-MHC) in HAVSMCs were inhibited on SF films or SFTSs, but expression could be obviously promoted by co-culture with HUVECs or HAFs, especially that of SM-MHC. On SF films, the expression of vascular endothelial growth factor (VEGF) and platelet endothelial cell adhesion molecule-1 (CD31) in HUVECs was promoted, and the expression levels of both increased obviously when co-cultured with HAVSMCs, with the expression levels of VEGF increasing with increasing incubation time. The expression levels of VEGF and CD31 in cells co-cultured on SFTSs improved significantly from day 3 compared with the mono-culture group. These results were beneficial to the mechanism analysis on vascular cell colonization and vascular tissue repair after in vivo transplantation of SFTSs.

## 1. Introduction

Cardiovascular disease caused by thrombosis or a vascular lesion is a major cause of death of in humans. The development of blood vessel substitutes has rapidly progressed in the past decade in response to the clinical need. Vascular grafts have been realized to replace large-diameter (inner diameter > 6 mm) blood vessels, and these have been made from materials such as Dacron^®^ [1] and e-polytetrafluoroethylene (e-PTFE) [2]. However, small-diameter (inner diameter < 6 mm) prosthetic vascular grafts for clinical applications remain unfeasible. Polyurethane [3], polyglycolic acid [4], polylactic acid [5], polycaprolactone [6] and their composites or derivatives [7,8] have been widely expected to be suitable for use as tissue-engineered vascular grafts prepared by freeze-drying [9], electrospinning [10], solvent casting and particulate leaching [11] and sintered microsphere [12]. Compared to synthetic materials, natural biopolymers are more conducive to cell adhesion, migration and proliferation and thus more suitable for biological material applications [13]. Collagen, hyaluronic acid and fibrin have been utilized to construct biological vascular grafts, resulting in good biocompatibility and promoting cell adhesion and differentiation [14,15,16]. However, the burst strength of these artificial vascular constructs is inferior to the best performing bypass grafts from a patient’s own blood vessel [17].

Cytocompatibility of materials affects the subsequent fate of tissue-engineered scaffolds [18]. Silk fibroin has been widely investigated as a biomaterial [19], due to its favorable cell compatibility [20,21,22], blood compatibility [23,24,25], adjustable biodegradation [24] and immunogenicity [26]. In a recent study, regenerated silk fibroin (SF) has been verified to support adhesion, migration and growth of vascular endothelial cells and smooth muscle cells in vitro [17,27]. The use of SF has been reported in fabricating artificial vessels prepared by electrospinning [28], salt leaching [29], or freeze-drying [17,27] and the biocompatibility of such vessels has been evaluated in vitro and in vivo. SF can be chemically modified by heparin grafting [30], sulfation [31], sulfonation [32] and hirudin-modification [25] to improve antithrombogenicity for small-diameter vascular grafts. Silk fabric has been considered in small-diameter artificial blood vessel fabrication to endow excellent mechanical properties and prevent collapse. In our previous work, a porous tubular scaffold prepared using silk fabric and regenerated SF, was able to support cell ingrowth and gradually degrade from a porous SF material finally to a silk fabric [33].

A blood vessel is a multicellular tissue, composed of three layers: endothelial tissue in the inner layer, smooth muscle tissue in the middle layer and connective tissue in the outer layer. The long-term patency of vascular tissue depends on the growth and physiologic responsiveness of the luminal endothelial layer [34]. Smooth muscle tissue composed of smooth muscle cells and extracellular matrix provides vascular mechanical properties which maintain the relaxation and contraction of blood vessels [35]. Elasticity and mechanical stability also depend on adventitia which is composed of fibroblasts contained within connective tissue. The physiological function and activity of vascular tissue depends on the co-growth and signal transduction of human aorta vascular smooth muscle cells (HAVSMCs) with human umbilical vein endothelial cells (HUVECs) and/or human arterial fibroblasts (HAFs). However, data are lacking on the response of different vascular cells to SF materials that relate to the successful development of tissue-engineered blood vessels [34]. An artificial vascular substitute would cause the infiltration of smooth muscle cells and endothelials cell after implantation in vivo. In order to achieve the application of silk fibroin small-diameter artificial blood vessels in clinic, it is necessary for us to study the interaction between different vascular cells on SF materials.

In the present study, vascular cells were co-cultured on SF films and SF tubular scaffolds (SFTSs) in order to evaluate the interaction between cells (HUVECs, HAVSMCs and HAFs). Cell morphology, cell proliferation and biological activity of HAVSMCs and HUVECs were explored.

## 2. Materials and Methods

### 2.1. Preparation of SF Solution

*Bombyx mori* SF solution was prepared as described previously [36]. Briefly, raw silk was boiled in 0.06 wt % Na_2_CO_3_ solution to remove sericin. After drying, degummed silk was dissolved in the ternary solvent CaCl_2_·CH_3_CH_2_OH·H_2_O (mole ratio 1:2:8) at a liquor ratio of 1:10 (*w*/*v*) at 70 ± 2 °C and stirred until completely dissolved. The mixed solution was dialyzed against distilled water at 4 °C for 3 days. The SF aqueous solution was concentrated to 8%.

### 2.2. Preparation of Regenerated SF Films and SFTSs

Insoluble SF matrix was obtained by chemical crosslinking with polyethylene glycol diglycidyl ether (PEG-DE, MW500D; Sigma, St. Louis, MO, USA). SF solution and PEG-DE were mixed together at a weight ratio of 1:0.8 with stirring for preparation of regenerated SF films and SFTSs. The mixed solution was cast onto a 24-well plate and air-dried at room temperature to prepare SF films. A silk fabric was braided by twisting 24 strands of degummed threads, which were twisted into 2 × 20/22 D yarns, on a braiding machine (Shanghai Hakao, Shanghai, China) at a speed of 160 rpm, and used to obtain a silk-knitted tube. The silk-knitted tube was dried in an oven at 60 °C after coating with a low concentration of SF solution and placed on a mold. The mixed solution of SF and PEG-DE was injected into the mold, then freeze-dried to form SFTSs.

The films and tubular scaffolds were immersed in deionized water for 3 days to remove unreacted molecules; then the tubular scaffolds were cut into small pieces that were placed carefully at the bottom of 24-well plates. Materials were used for subsequent experiments after sterilizing by C_o_^60^ gamma irradiation.

### 2.3. Cell Culture

Human arterial fibroblasts (HAFs; ATCC, Manassas, VA, USA) and human umbilical vein endothelial cells (HUVECs; ATCC) were cultured in Dulbecco’s Modified Eagles Medium (Gibco, Carlsbad, CA, USA) and human aorta vascular smooth muscle cells (HAVSMCs; ATCC) were cultured in Roswell Park Memorial Institute 1640 Medium (Gibco). Both culture media contained 10% fetal bovine serum (Gibco), 100 U/mL penicillin, and 100 μg/mL streptomycin, at 37 °C in a 5% CO_2_ incubator. During the logarithmic growth phase, cells were trypsinized using 0.25% trypsin (Sigma) and resuspended in fresh medium with fetal bovine serum and antibiotics. Then 1.5 × 10^4^ cells in single-cultured groups (HAFs, HUVECs, HAVSMCs and control group) were added to each well of a 24-well plate with SF films and cultured at 37 °C in a 5% CO_2_ incubator, and cells in co-cultured groups (HUVECs/HAVSMCs or HAFs/HAVSMCs) were added to a 24-well plate at 1:1 radio, consisting of 7.5 × 10^3^ cells of each type. Cell morphology was observed using an IX51 inverted microscope (Olympus, Tokyo, Japan) at day 1 and day 5. DNA content was determined at day 1, 3, 5 and 7. Half of the medium was replaced every other day.

SFTSs in 24-well plates were immersed in Dulbecco’s Modified Eagles Medium for 24 h at 4 °C. A suspension of HAVSMCs (2 × 10^5^ cells) was injected into the interior of scaffolds through the cross-section of the scaffold, then the 24-well plate was placed in a 5% CO_2_ incubator for 2 h. A suspension of HUVECs (2 × 10^5^ cells) was evenly added to the inner surface of the scaffolds seeded with HAVSMCs. The seeded 24-well plates were fed with medium and cultured in a 5% CO_2_ incubator. The medium was replaced every other day.

### 2.4. DNA Extraction and DNA Content Determination

Cells in each well were collected after digestion with 0.25% trypsin and centrifuged at 1500× g for 15 min. Total DNA was extracted according to the instructions of the low dose genomic DNA extraction kit (Beyotime Institute of Biotechnology, Haimen City, China) using spin columns, and the absorbance at 280 nm (*A*_280_) and 260 nm (*A*_260_) was measured using a SmartSpec Plus UV/Vis spectrophotometer (Bio-Rad, Hercules, CA, USA). DNA was re-extracted if the *A*_260_/*A*_280_ ratio was less than 1.8. DNA content was calculated according to the following equation:

DNA content (μg) = *a* × *b* × 50 × *V*, (1)

where *a* is the measured value of *A*_260_, *b* is the dilution factor, and *V* is the total volume (mL).

### 2.5. Immunofluorescence Analysis

Cells at day 1, 3 and 5 were fixed at room temperature for 30 min with 4% paraformaldehyde and permeabilized with 0.2% Triton X-100 in phosphate-buffered saline (PBS, pH = 7.4), followed by blocking with 2% bovine aerum albumin for 30 min at room temperature. Samples were incubated overnight at 4 °C with 200 μL of mouse anti-human α-SMA primary antibody (Sigma) and rabbit anti-human CD31 primary antibody (Abcam, Cambridge, UK). After washing 3 times with PBS (pH = 7.4), samples were reacted with goat anti-mouse IgG secondary antibody (Alexa Fluor^®^ 488, Abcam) or anti-rabbit IgG secondary antibody (Alexa Fluor^®^ 594 Goat, Life Technology, Carlsbad, CA, USA) for 2 h at room temperature, then 1 mL (5 μg/mL) of 4',6-diamidino-2-phenylindole (DAPI) was added into each well to stain the nuclei at room temperature for 15 min, then washed with PBS (pH = 7.4) to remove excess DAPI dyestuff. Fluorescently-labelled cells were observed at 488 nm using an FV1000 confocal laser scanning microscope (Olympus).

### 2.6. SEM Analysis

The morphology of cells co-cultured on SFTSs was examined by scanning electron microscopy (SEM) using a Hitachi S-4700 (Hitachi, Tokyo, Japan). After incubation for 1, 3, or 5 days, SFTSs were washed twice with PBS (pH = 7.4) and fixed in 2.5% glutaraldehyde for 12 h at 4 °C, then washed again with PBS (pH = 7.4). The samples were dehydrated by exposing to a gradient concentration of ethanol (30, 50, 80 and 100%) for 10 min each. All samples were coated with gold and observed by SEM.

### 2.7. RT-PCR

Total RNA was extracted from cells at day 1, 3, 5 and 7 using a total RNA Purification Kit (Sangon Biotech, Shanghai, China) following the supplier’s instructions. Then samples were treated with an RNA Cleanup and Concentration Kit (Sangon Biotech) to ensure RNA samples were free from contaminating DNA. RNA was subjected to reverse transcription using a Moloney murine leukemia virus (M-MuLV) first chain cDNA synthesis Kit (Sangon Biotech). Quantitative real-time PCR was carried out using a Step One Plus Real-Time PCR System (Applied Biosystems, Foster City, CA, USA) with Power SYBR Green PCR Master Mix (Applied Biosystems). PCR cycling conditions were: pre-denaturation at 95 °C for 15 min, followed by 40 cycles of denaturation at 95 °C for 30 s, annealing at the appropriate temperature (54–60 °C; Table 1) for 30 s and elongation at 72 °C for 30 s. Genes of CD31, VEGF for HUVECs and SM-MHC and α-SMA for HAVSMCs were selected and the human housekeeping gene of glyceraldehyde-3-phosphate dehydrogenase (GAPDH) was used as the reference transcript. Primer sequences are shown in Table 1. The 2^−^^△△CT^ method was used for quantitative analysis. Each sample was analyzed in triplicate.

### 2.8. DNA Agarose Gel Electrophoresis

Agarose (1.5 g) was dissolved in 100 mL TBE (54 g Tris base, 27.5 g boric acid, 4.14 g ethylene diamine tetraacetic acid, pH = 8.0–8.2) by heating for 2 min, then mixed with 10 μL Gel Red™ nucleic acid gel stain (Biotium, Fremont, CA, USA) to obtain a 1.5% agarose gel. A 10 μL aliquot of the product of primer amplification was mixed with 2 μL gel loading buffer (Sigma), then the mixture and 10 μL DNA marker (50 bp DNA Ladder, Takara Bio Inc., Shiga, Japan) were added into the loading wells of the gel. Gel electrophoresis was performed on a nucleic acid electrophoresis apparatus (BIO-RAD) at 100 V for 90 min. Finally, the gel was photographed using a Canon digital camera (EOS50D, Canon, Tokyo, Japan).

### 2.9. Statistical Analysis

Data are presented as mean ± standard error of the mean. Comparisons of means were performed using one-way analysis of variance, followed by an independent sample *t*-test using SPSS 17.0 statistical software (IBM, Armonk, NY, USA). Results with *p* < 0.05 were considered to be statistically significant.

## 3. Results and Discussion

### 3.1. Cell Morphology and Proliferation of HAVSMC/HUVEC Co-Cultures

**Figure 1:**
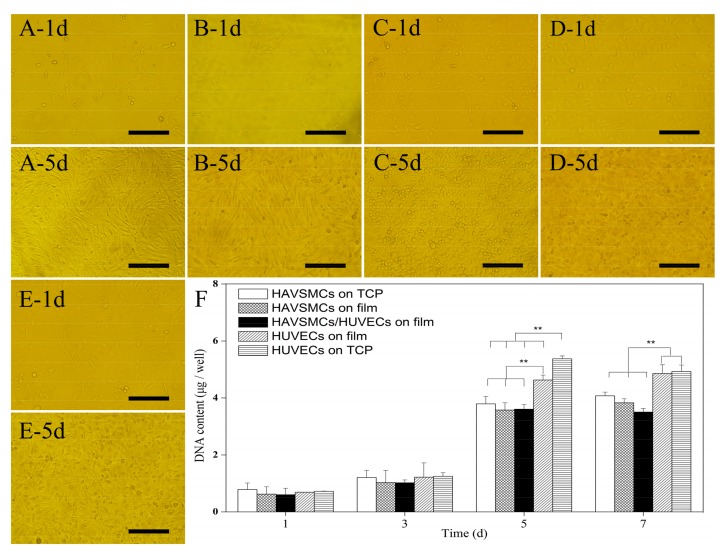
Cell morphology and cell proliferation of human aorta vascular smooth muscle cells (HAVSMCs)/human umbilical vein endothelial cells (HUVECs) co-cultured on silk fibroin (SF) films. (**A**) HAVSMCs on tissue culture plate (TCP); (**B**) HAVSMCs on film; (**C**) HUVECs on TCP; (**D**) HUVECs on film; (**E**) HAVSMC/HUVEC co-cultures on film and (**F**) DNA contents, Bar = 200 μm, ** *p* < 0.01.

HUVECs and HAVSMCs are the main constituent cells of blood vessels and are closely related in structure and function, with the cells’ biological characteristics influencing each other by cell signal transduction. Figure 1 shows the cell morphology and cell proliferation of co-cultured HUVECs/HAVSMCs on SF films. Cells spread uniformly on films and the cell density gradually increased from day 1 to day 5. Cells of HAVSMCs, HUVECs or co-cultures were all fully confluent on SF films after 5 days, with HAVSMCs exhibiting a spindle shaped morphology (Figure 1A,B), and HUVECs spread in a typical pebble shape (Figure 1C,D) on film and tissue culture plate (TCP). Spindle-shaped HAVSMCs and pebble-shaped HUVECs were alternately and evenly distributed on co-cultured SF films (Figure 1E). The results indicated that HAVSMCs and HUVECs could coexist on SF films without rejection or separation.

Figure 1F shows the DNA contents of co-cultured HAVSMCs/HUVECs on SF films. The DNA content of HAVSMCs and HUVECs increased in all samples, but the increase was relatively slow from day 1 to day 3. By 5 days after seeding, the DNA content of cells had increased significantly from 0.6–0.8 μg/well to 3.5–5.4 μg/well. As can be seen from Figure 1F, HUVECs proliferated significantly faster than HAVSMCs under the same conditions either on SF film or on TCP, and the DNA content was also significantly higher than that of co-cultured HAVSMCs/HUVECs. HAVSMCs spread into long spindle and interconnected into a network structure. Thus, there was insufficient interface remaining for cell proliferation, while plenty of space between cells, where cells are difficult to access. Therefore, there was no significant difference in the DNA content in groups of HAVSMCs between those on TCP, in mono-culture or in co-culture on SF films.

### 3.2. Cell Morphology and Proliferation of HAVSMC/HAF Co-Cultures

**Figure 2:**
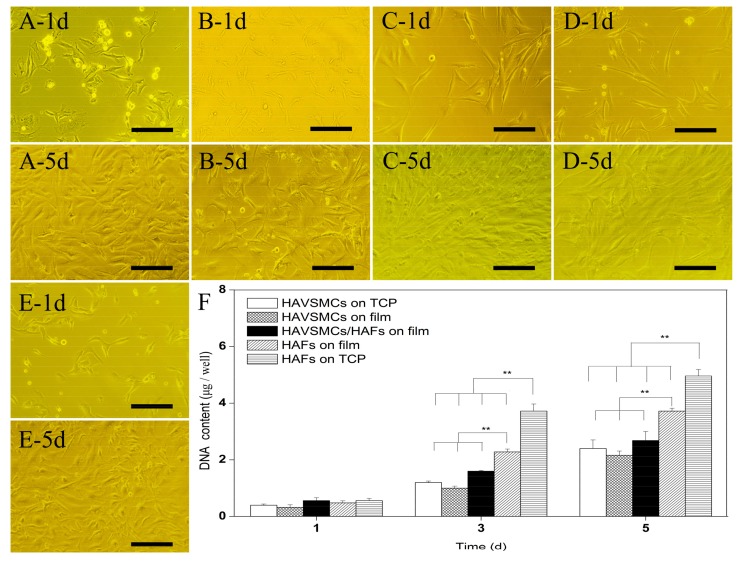
Cell morphology and cell proliferation of HAVSMCs/human arterial fibroblasts (HAFs) co-cultured on SF films. (**A**) HAVSMCs on TCP; (**B**) HAVSMCs on films; (**C**) HAFs on TCP; (**D**)HAFs on films; (**E**) HAVSMC/HAF co-cultures on films and (**F**) DNA contents, Bar = 200 μm, ** *p* < 0.01.

Cell morphology of HAVSMC/HAF co-cultures on SF films is shown in Figure 2. At 1 day after seeding, HAVSMCs and HAFs had adhered and spread into a typical thread shape. HAVSMCs extended multiple pseudopodia, while HAFs exhibited a longer spindle shape (Figure 2A–D). When day 1 was compared with day 5, HAVSMCs and HAFs all showed strong cell proliferation on SF films, including co-cultured HAVSMCs/HAFs. Cells co-cultured on SF film spread well from day 1 to day 5 after seeding (Figure 2E).

Figure 2F shows the DNA content of HAVSMCs and HAFs grown on SF films. The DNA content of all samples was similar on day 1 and had obviously increased by day 3 after seeding. The DNA content of HAFs on TCP was significantly higher than that on SF films, with an increase of up to 1.39-fold. When the three groups on SF films were compared, the DNA content of HAFs was the highest, followed by the HAVSMC/HAF co-cultures. The DNA content on the fifth day continued to increase on all SF films, including the HAVSMC/HAF co-cultures. Analysis of DNA content showed that SF films obviously inhibited the proliferation of HAFs; however, the proliferation rate of HAFs was significantly higher than that of HAVSMCs cultured either on TCP or on SF films.

### 3.3. Immunofluorescence Analysis of Co-Cultured Cells

**Figure 3:**
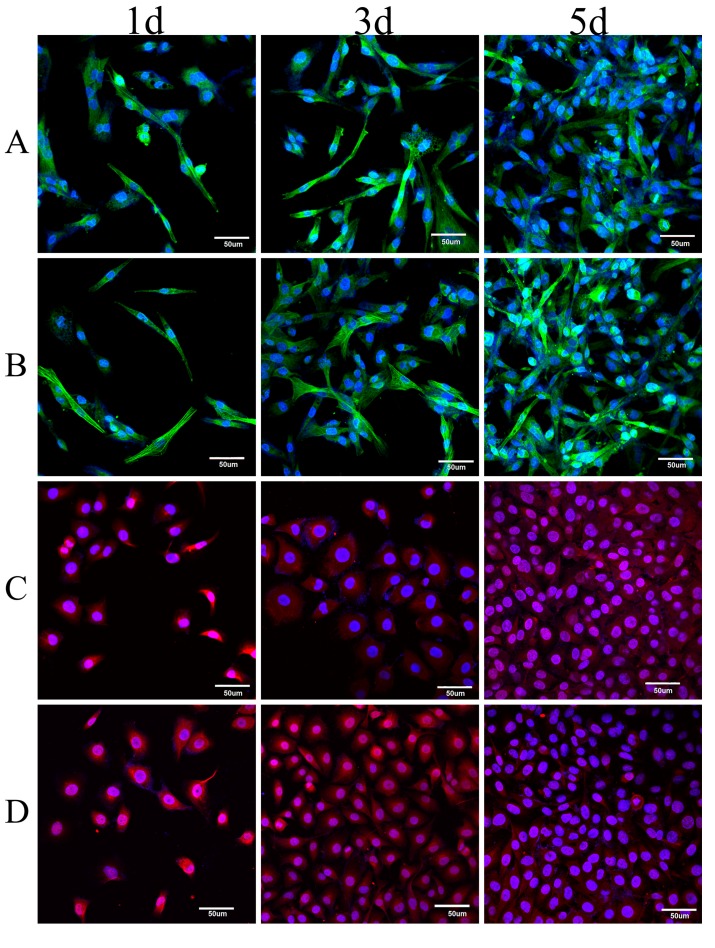

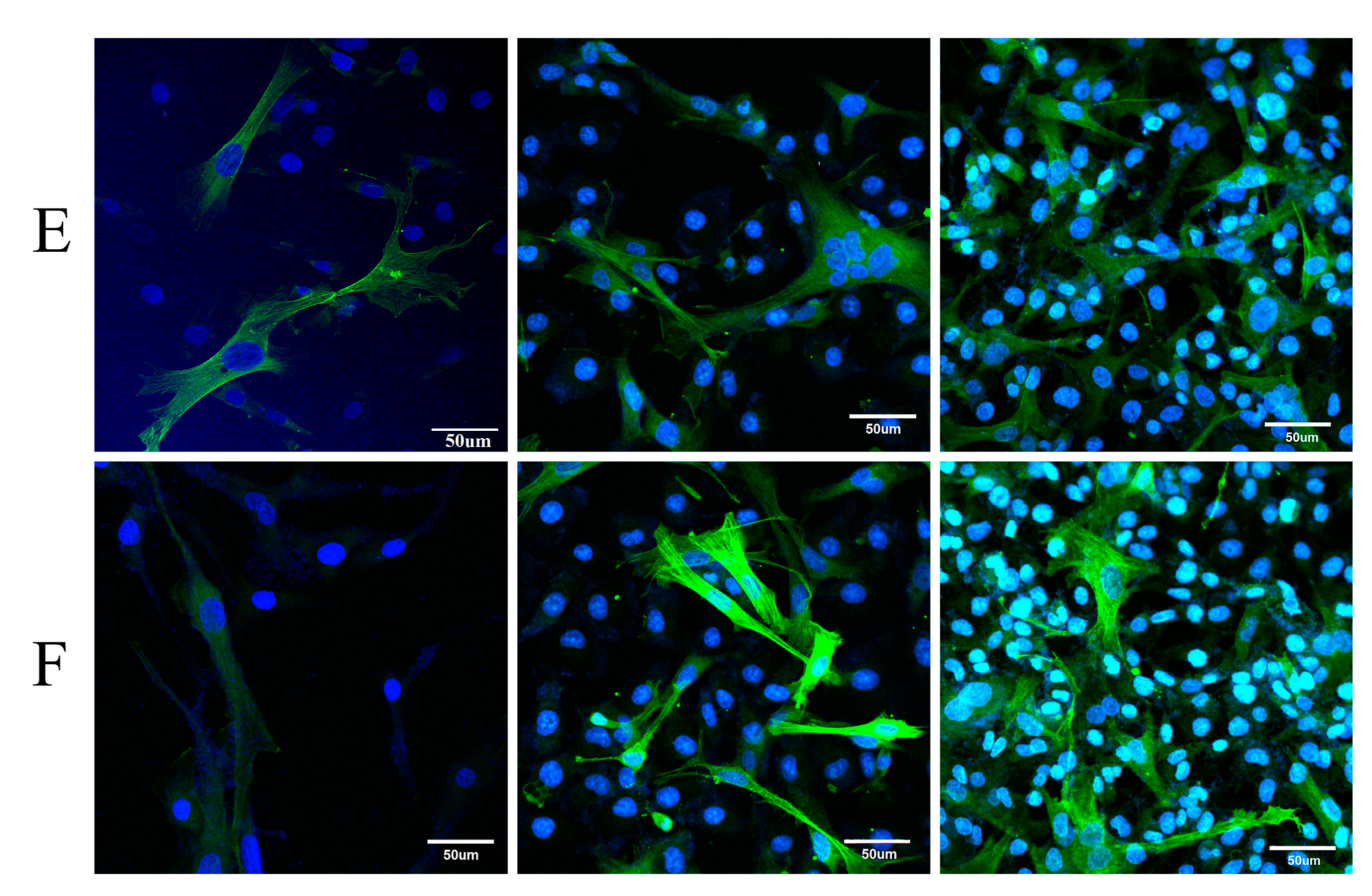
Immunofluorescence of HAVSMCs co-cultured with HUVECs or HAFs on SF films. Green was for α-SMA in HAVSMCs, blue was for cell nucleus and red was for CD31 in HUVECs, (**A**) HAVSMCs on films; (**B**) HAVSMCs on TCP; (**C**) HUVECs on films; (**D**) HUVECs on TCP; (**E**) HUVEC/HAVSMC co-cultures on film and (**F**) HAF/HAVSMC co-cultures on film, Bar = 50 μm.

Figure 3 shows the immunofluorescence of vascular cells after culture for 1, 3 or 5 days. The numbers of HAVSMCs and HUVECs cultured on SF films continuously increased from day 1 to day 3, showing high cellular proliferative activity (Figure 3A,C), which was comparable to that on TCP (Figure 3B,D). When co-cultures were stained, the green fluorescence of α-SMA indicated only the HAVSMCs while the blue fluorescent nuclei represented both types of cells in co-culture. As shown in Figure 3E,F, co-cultured cells fully and evenly spread on the films after 5 days, both of the fluorescence intensities on day 5 were significantly stronger than on day 1. The blue fluorescence intensity of co-cultured HUVECs/HAVSMCs on SF film was the same as that of HUVECs or HAVSMCs cultured alone on SF films. The same result was observed in co-cultured HAVSMCs/HAFs. As can be seen from Figure 3, HAVSMCs spread very well whether cultured alone or co-cultured on SF film.

Combining the results of immunofluorescence photographs and DNA contents, it was apparent that HUVECs and HAFs were in the dominant position when co-cultured with HAVSMCs. Intimal hyperplasia caused by HAVSMCs over-proliferation is not only an important factor in the formation of a thrombus after vascular grafting [37], but also the key reason that the creation of small-diameter artificial blood vessels for clinical application has not yet been achieved. Our results indicated that SF materials can spontaneously regulate the different growth characteristics of vascular cells in co-culture, promoting endothelialization and preventing intimal hyperplasia.

### 3.4. Cell Morphology on SFTSs

**Figure 4:**
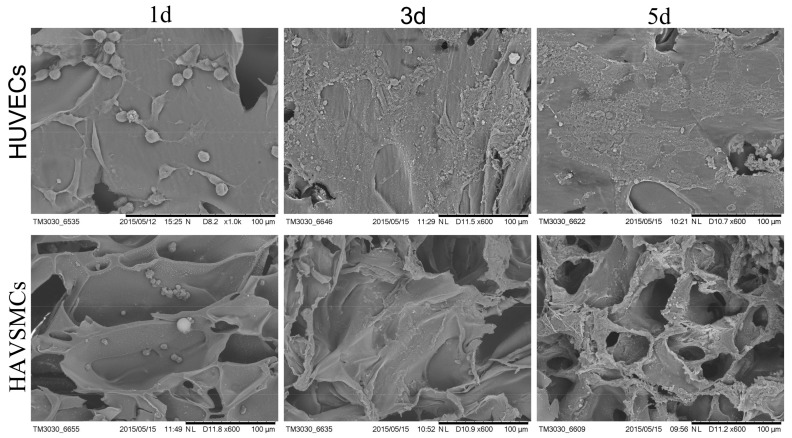
SEM photographs of HUVECs and HAVSMCs on SFTSs. Bar = 100 μm.

HUVECs were implanted on the inner surface of SF tubular scaffolds, and HAVSMCs were implanted in the pores of the scaffold walls. SEM micrographs of HUVECs and HAVSMCs on SFTSs are shown in Figure 4. The results showed that the HUVECs adhered and spread well on the inner surface of SFTSs on the first day of seeding, while HAVSMCs adhered to the pore walls in SFTSs. The cell number of both cell types increased on the third and fifth day of culture. From day 3, HUVECs spread in a pebble shape and interconnected via the extracellular matrix. On day 5, HAVSMCs were spread over the walls of the internal pores in SFTSs. SEM photographs showed that both HUVECs and HAVSMCs preferred to adhere and spread well, and had high proliferative activity on SFTSs. Moreover, both cell types connected with each other and were embedded in a great deal of cellular matrix.

### 3.5. Appraisal of Primers for PCR

**Figure 5:**
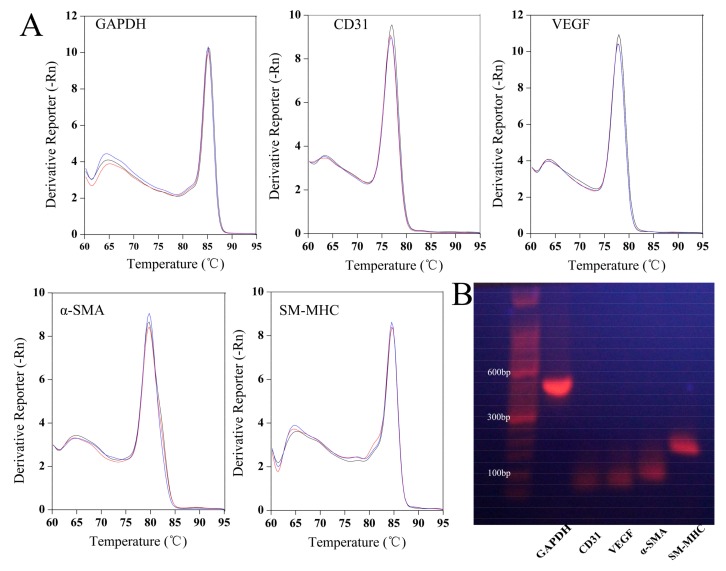
Specific identification of each primer. (**A**) Melting curve and (**B**) DNA agarose gel electrophoresis. Different colors in the figure A represent the different samples.

Primers used for analysis need to be specific. Figure 5 shows the melting curves and DNA agarose gel electrophoresis of primers for GAPDH, CD31, VEGF, α-SMA and SM-MHC. The melting curves of each primer all showed a single peak (Figure 5A). The amplification efficiencies of GAPDH, CD31, VEGF, α-SMA and SM-MHC were 105.677, 108.635, 108.152, 106.284 and 90.112% (Table 1), respectively. DNA agarose gel electrophoresis shows that each primer amplified products that generated a clear single band and the amplified band size was shown to be consistent with the theoretical value according to the DNA molecular weight standards, which were 420, 67, 80, 88, and 158 bp, respectively for GAPDH, CD31, VEGF, α-SMA and SM-MHC (Figure 5B). The results of agarose gel electrophoresis and melting curve analysis indicated that each primer for amplification of GAPDH, CD31, VEGF, α-SMA and SM-MHC was specific.

### 3.6. Cytokine Expression Levels of Co-Culture on SF Films

#### 3.6.1. HUVEC/HAVSMC Co-Cultures

**Figure 6:**
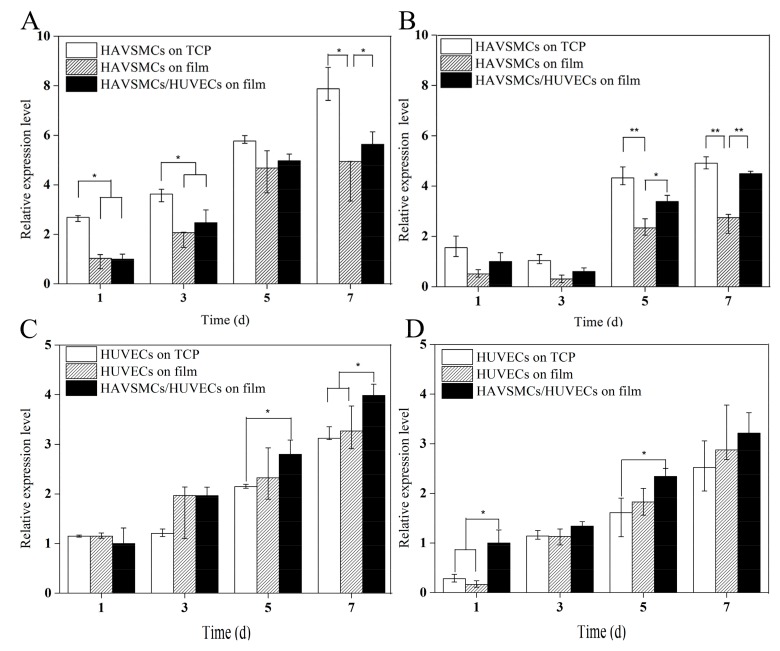
Cytokine expression of HUVECs/HAVSMCs co-cultured on SF films. (**A**) α-SMA; (**B**) SM-MHC; (**C**) VEGF and (**D**) CD31, * represents *p* < 0.05, ** represents *p* < 0.01.

The relative expression levels of the cytokines α-SMA, SM-MHC, VEGF and CD31 in HUVECs/HAVSMCs co-cultured on SF films are shown in Figure 6. During the incubation period, the α-SMA expression levels in HAVSMCs cultured on all samples increased significantly (Figure 6A). The relative expression level of α-SMA on day 5 was 2.2–4.6 times as high as that on day 1. At the beginning of culture, the α-SMA expression level of co-cultured HUVECs/HAVSMCs on SF films was close to that of mono-cultured HAVSMCs on SF films. On day 3 and on SF films, the relative expression level of α-SMA of co-cultured HAVSMCs was slightly higher than that of mono-cultured HAVSMCs, with increases of up to 1.2-fold. The change in the expression level of SM-MHC in HAVSMCs on each sample was similar to the changes in α-SMA (Figure 6B). The expression of α-SMA and SM-MHC in HAVSMCs was inhibited when cultured on SF films compared with TCP, but was increased by co-culture with HUVECs, especially the expression of SM-MHC. On SF films, the relative expression level of SM-MHC in co-cultured HAVSMCs was significantly higher than that of mono-cultured HAVSMCs.

In contrast, instead of being inhibited, the expression of both VEGF and CD31, the specific markers in HUVECs, was slightly improved when cultured on SF films (Figure 6C,D). The expression levels of both VEGF and CD31 increased continuously with time when cultured on all samples from day 1 to day 7. On day 3, there was no significant difference in the expression levels of VEGF and CD31 in co-cultured HUVECs compared with mono-cultured cells on SF film, but the expression level of VEGF in co-cultured HUVECs was obviously higher on day 5 and significantly higher on day 7 than that of mono-cultured HUVECs. The expression level of CD31 in co-cultured HUVECs was higher than that of mono-cultured HUVECs on SF film, and significantly higher than that of cells on TCP on day 5. Results indicated that SF films were the best for growth of HUVECs, and co-cultures of HUVECs/HAVSMCs on SF films could promote the expression of the cytokines α-SMA, SM-MHC, VEGF and CD31.

#### 3.6.2. HAVSMC/HAF Co-Cultures

**Figure 7:**
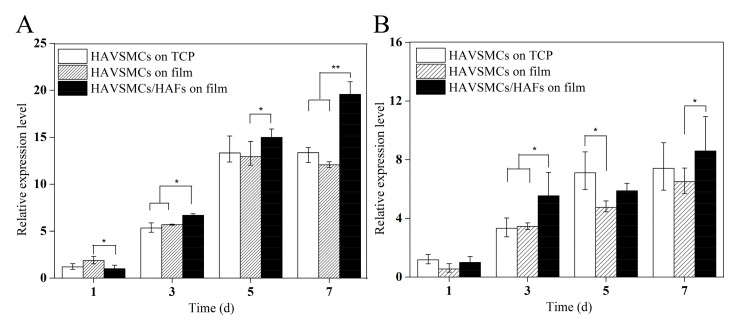
Cytokine expression of HAVSMCs/HAFs co-cultured on SF films. (**A**) α-SMA and (**B**) SM-MHC, * *p* < 0.05, ** *p* < 0.01.

As shown in Figure 7, the α-SMA expression of each sample significantly increased with time, with the expression levels of α-SMA on days 3 and 5 reaching more than 3 times and 6 times that on day 1 (Figure 7A). The relative expression level of α-SMA in co-cultured HAVSMCs was higher than that in the other two groups, especially on day 7 when it showed a significant difference. The expression level of SM-MHC in each sample was also significantly increased on day 3 (Figure 7B). Compared with mono-cultures on SF films, the relative expression level of SM-MHC in co-cultured HAVSMCs was significantly higher at 3 days after seeding. Results showed that the expression of α-SMA and SM-MHC in HAVSMCs cultured on SF film was promoted by co-culture with HAFs, even though SF films had a slight inhibitory effect on their expression.

### 3.7. Cytokine Expression Levels of Co-Culture on SFTSs

#### 3.7.1. HUVEC/HAVSMC Co-Cultures

**Figure 8:**
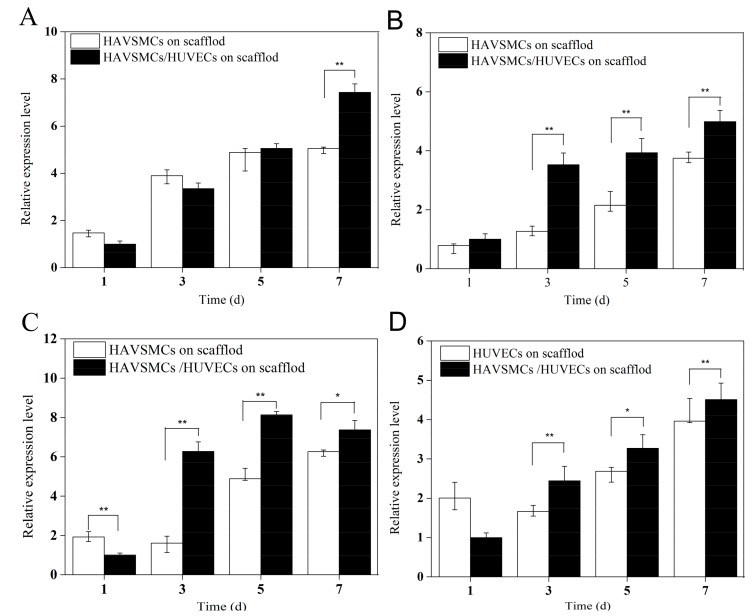
Cytokine expression of HUVECs/HAVSMCs co-cultured on SFTSs. (**A**) α-SMA; (**B**) SM-MHC; (**C**) VEGF and (**D**) CD31, * *p* < 0.05, ** *p* < 0.01.

Rapid adhesion and proliferation of endothelial cells is crucial to endothelialization after artificial vascular grafting, preventing thrombosis and maintaining long-term patency [38]. There is increasing evidence that cell-to-cell interactions can control cellular growth, migration, differentiation, and function. Porous 3D structure is more conducive to signal transduction between cells. In order to thoroughly explore and analyze the tissue reconstruction of multicellular blood vessels after their replacement with SF scaffolds, we conducted a study on co-culture of HUVECs/HAVSMCs and HAVSMCs/HAFs on regenerated porous SFTSs in vitro. Figure 8 shows the cytokine expression of co-cultured HUVECs/HAVSMCs on 3D scaffolds. The expression levels of α-SMA (Figure 8A) and SM-MHC (Figure 8B) both increased obviously from day 1 to day 7, but the effect of co-culture was delayed on α-SMA. When culture was extended to day 5, there was no obvious difference in α-SMA expression between co-cultures and mono-cultures on 3D scaffolds, but the expression level was significantly higher in co-cultures compared to mono-cultures on day 7. Co-culture on 3D scaffolds had a significant effect on the expression of SM-MHC, and the expression level was significantly higher than that in mono-culture on day 3, with an increase of up to 3-fold.

The expression of VEGF and CD31 in the co-culture group also increased obviously with time, and both were significantly higher than that of the mono-culture groups after 3 days (Figure 8C,D). Compared with mono-cultures of HUVECs, the expression levels of VEGF and CD31 in the co-cultured group were increased 3.92-fold and 1.47-fold, respectively, on day 3, and 1.66-fold and 1.22-fold, respectively, on day 5. Results showed that co-culture of HUVECs/HAVSMCs on 3D SF scaffolds significantly enhanced cytokine expression, especially expression of SM-MHC, VEGF and CD31.

#### 3.7.2. HAVSMC/HAF Co-Cultures

**Figure 9:**
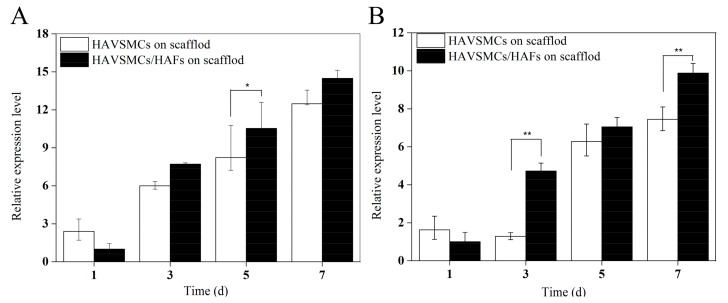
Cytokine expression of HAVSMCs/HAFs co-cultured on SFFSs. (**A**) α-SMA and (**B**) SM-MHC, * *p* < 0.05, ** *p* < 0.01.

Cytokine expression of HAVSMCs/HAFs co-cultured on SFFSs is shown in Figure 9. The expression levels of α-SMA and SM-MHC on the scaffolds increased gradually over the culture period. The expression of α-SMA in the co-culture group was lower than that in the mono-culture group on the first day, but the difference was not significant (Figure 9A). The expression levels of α-SMA on day 5 and of SM-MHC on day 3 in co-culture were significantly higher than in the mono-culture group (Figure 9B). Thus, co-culture with HAFs on STSFs enhanced the expression of α-SMA and SM-MHC in HAVSMCs.

## 4. Conclusions

The results of cell morphology, DNA content and immunofluorescence analysis showed that SF materials could be a good substrate for the adhesion, spreading and proliferation of HUVECs, HAVSMCs and HAFs. SF materials favored for the growth of HUVECs leading to improve expression of VEGF and CD31, and their expression levels were significantly enhanced by co-culture with HAVSMCs. The expression of α-SMA and SM-MHC in HAVSMCs cultured on SF materials was promoted by co-culture with HUVECs or HAFs, although SF materials had a slight inhibitory effect on their expression. In addition, HAVSMCs on SF materials proliferated more slowly than HAFs and HUVECs, which could help to prevent intimal hyperplasia, one of the major barriers to tissue repair using artificial blood vessels. This study explored the biological characteristics of vascular cells through co-culture in vitro. Results were beneficial to the mechanism analysis on endothelialization, cell infiltration and vascular tissue repair using silk fibroin tubular scaffolds in vivo.

## Figures and Tables

**Table 1:** The primer sequences, product length, annealing temperature, and amplification efficiency of each cytokine.

Gene	Primer Sequence (5′–3′)	Product Length (bp)	Annealing Temperature (°C)	Amplification Efficiency (%)
GAPDH	GTCACTGGTGGACCTGACCTAGGGGTCTACATGGCAACTG	420	60	105.677
VEGF	GCTCAGAGCGGAGAAAGCATGCAACGCGAGTCTGTGTTTT	80	54	108.152
CD31	GGTGGATGAGGTCCAGATTTCCAGCACAATGTCCTCTCCAG	67	56	108.635
α-SMA	CCGACCGAATGCAGAAGGAACAGAGTATTTGCGCTCCGGA	88	56	106.284
SM-MHC	GGCCGTCAAGTCCAAGTTCAACCACCTGCAGCAAGATTTCC	158	56	90.112
